# Metabolomic Biomarkers for Monitoring Tuberculosis Treatment Response: A Comprehensive Literature Review

**DOI:** 10.3390/diagnostics16091278

**Published:** 2026-04-23

**Authors:** Hien Thi Thu Nguyen, Tuong Khanh Bui-Nguyen, Chi Que Nguyen, Hanh Thi My Dinh, Trang Khanh Tran, Nhung Thi Thuy Hoang, Huong Minh Nguyen, Vang Le-Quy, Alexei Korobitsyn, Linh Nhat Nguyen

**Affiliations:** 1 Department of Molecular Diagnostics, Aalborg University Hospital, 9260 Aalborg, Denmark; 2 Department of Clinical Medicine, Aalborg University, 9260 Aalborg, Denmark; 3 AVSE Global Medical Translational Research Network, 75008 Paris, France; ktuong8008@gmail.com (T.K.B.-N.); quechi.nguyen@avseglobal.org (C.Q.N.); dinh.myhanh09@gmail.com (H.T.M.D.);; 4 Department of Pharmacy, Becamex International Hospital, Ho Chi Minh 75206, Vietnam; 5 Master Program in Smart Healthcare Management, International College of Sustainability Innovations, National Taipei University, New Taipei City 23741, Taiwan; 6 Faculty of Life Sciences: Food, Nutrition and Health, University of Bayreuth, 95326 Bayreuth, Germany; 7 Novodan ApS, 9220 Aalborg, Denmark; 8 AVSE Global Data Science Network, 75008 Paris, France; 9 Department for HIV, Tuberculosis, Hepatitis and Sexually Transmitted Infections, World Health Organization (WHO), 1211 Geneva, Switzerland

**Keywords:** tuberculosis, metabolomics, biomarkers, treatment outcome, treatment response

## Abstract

Tuberculosis (TB) remains a major global cause of morbidity and mortality. Current tools for monitoring treatment response rely on sputum-based microscopy and culture, which are often insensitive, time-consuming, and impractical in extrapulmonary or pediatric TB and in individuals unable to produce sputum. Metabolomics has emerged as a promising approach for identifying host-derived biomarkers that reflect treatment-associated immunometabolic changes; however, the available evidence remains heterogeneous and has not been comprehensively synthesized. We conducted a comprehensive literature review of human studies evaluating metabolomic biomarkers in relation to TB treatment response or outcomes. PubMed, Scopus, and EMBASE were searched for human studies evaluating targeted or untargeted metabolomics (NMR, LC-MS, GC-MS, CE-MS) in relation to treatment response or outcomes. Two reviewers independently screened studies, extracted data, and assessed risk of bias using QUIPS and PROBAST. Findings were synthesized using a structured framework organized across treatment stages and outcomes. Of 218 records identified, 139 titles and abstracts were screened and 42 full texts assessed; 15 studies met the inclusion criteria. Recurrent treatment-associated signals involved amino acid metabolism, particularly the tryptophan–kynurenine pathway, as well as vitamin and cofactor metabolites (pyridoxate, nicotinamide, trigonelline). Plasma studies frequently reported lipid remodeling and bile acid perturbations, whereas urine studies highlighted polyamine metabolism (e.g., N^1^,N^12^-diacetylspermine) and fatty acid β-oxidation markers. Common limitations included inadequate adjustment for confounders and, in prediction models, small sample sizes and limited external validation. Metabolomics reveals reproducible but heterogeneous immunometabolic changes during TB therapy. Key pathways include tryptophan–kynurenine metabolism, vitamin and cofactor metabolism, lipid remodeling, and urine polyamine pathways. Standardization and prospective multicenter validation are needed for clinical translation.

## 1. Introduction

Tuberculosis (TB) remains one of the leading causes of death from infectious disease worldwide, with an estimated 10.7 million new cases and 1.23 million deaths reported in 2024 [1,2]. Although effective treatment regimens exist, timely and accurate monitoring of treatment response remains a major clinical and public health challenge, particularly in settings with limited laboratory infrastructure [1,3,4,5,6,7]. Reliable treatment monitoring is essential for confirming cure, detecting non-response outcomes, and identifying individuals at risk of relapse or treatment failure.

Current tools for monitoring TB treatment response rely primarily on sputum smear microscopy and mycobacterial culture. However, smear microscopy lacks sensitivity and does not reliably reflect bacterial clearance, while culture is time consuming, resource-intensive, and unavailable in all routine care settings [1,3,8,9]. These limitations are particularly evident in individuals who cannot expectorate sputum, in those with extrapulmonary TB, and in children and adolescents, for whom microbiological confirmation and treatment monitoring are especially challenging. As a result, there is strong interest in host-based biomarkers that can provide earlier and more reliable indicators of treatment response across diverse patient populations.

In this context, metabolomics offers a promising approach for biomarker discovery by enabling comprehensive profiling of small molecules that reflect host immune activation, inflammation, tissue remodeling, and metabolic recovery during therapy [10,11,12,13,14,15]. Because TB is characterized by complex host–pathogen interactions, metabolomic signatures may capture both systemic immunometabolic perturbations and treatment-associated normalization across multiple biological pathways. Several studies have reported candidate metabolite or multimetabolite signatures associated with treatment response indicators, including early microbiological response (e.g., culture conversion) as well as final treatment outcomes such as treatment success, failure or relapse, using diverse biospecimens (e.g., plasma or urine) and analytical platforms (e.g., liquid chromatography–mass spectrometry (LC-MS), gas chromatography–mass spectrometry (GC-MS), nuclear magnetic resonance (NMR), and capillary electrophoresis–mass spectrometry (CE–MS) [16,17,18,19,20,21,22,23,24,25,26,27,28,29,30].

Despite these promising findings, the available evidence base remains heterogeneous. Studies differ substantially in biospecimen type, metabolomic workflows, sampling schedules, metabolite identification confidence levels, statistical modeling strategies, and definitions of treatment response. In addition, key clinical factors such as HIV infection and diabetes mellitus, both of which influence metabolic pathways implicated in TB, are inconsistently represented across cohorts, complicating comparisons across studies. This variability complicates comparison across studies and currently constrains the translation of metabolomic biomarkers into clinically actionable monitoring tools [31].

To address these gaps, we conducted a comprehensive literature review to synthesize metabolomic biomarkers associated with TB treatment response. This study follows PRISMA reporting guidance. While previous reviews summarized metabolomic alterations in active TB, only a few specifically examined metabolomic changes linked to treatment dynamics and clinical outcomes during therapy. In this review, we focused on human studies using targeted or untargeted metabolomic approaches to investigate both longitudinal metabolic changes during treatment and metabolites measured at treatment initiation that may predict subsequent outcomes such as treatment failure or relapse. By integrating evidence across studies, we aimed to identify recurrent metabolites and biological pathways associated with treatment response and to highlight promising candidates for future validation and translation to clinical practice. Compared with previous reviews, this study provides a structured synthesis across treatment stages, biospecimens, and outcome categories, enabling identification of recurrent pathway-level signals relevant to clinical translation.

## 2. Materials and Methods

### 2.1. Literature Search Strategy and Study Selection

This study was conducted as a comprehensive literature review, using structured search and study selection procedures informed by Preferred Reporting Items for Systematic Reviews and Meta-Analyses (PRISMA) reporting guidelines [32]. The review protocol was prospectively registered in the International Prospective Register of Systematic Reviews (PROSPERO) under registration number CRD420251266516. The objective was to synthesize evidence on metabolomic biomarkers associated with TB treatment response by examining both longitudinal changes across treatment stages (e.g., baseline, intensive phase, and end of treatment) and metabolite signatures associated with treatment outcomes such as treatment failure or relapse.

### 2.2. Eligibility Criteria (PECOS Framework)

#### 2.2.1. Population

We included studies involving human participants of any age with microbiologically or clinically diagnosed TB (pulmonary or extrapulmonary), including drug-susceptible and drug-resistant TB. Studies enrolling HIV-positive and/or HIV-negative participants were eligible. Animal and in vitro studies were excluded.

#### 2.2.2. Exposure/Intervention

We included studies using targeted or untargeted metabolomics, including NMR, LC–MS, GC–MS, and CE–MS platforms. Lipidomics studies were included when metabolite-level biomarkers were reported.

#### 2.2.3. Comparators

Eligible comparisons included treatment responders versus non-responders; culture converters versus non-converters; and treatment success versus treatment failure or relapse. We also included longitudinal within-person comparisons during therapy (e.g., baseline vs. intensive phase and/or baseline vs. end of treatment). Studies focusing solely on TB diagnosis (TB vs. non-TB controls) without treatment monitoring or treatment-outcome prediction were not included in the treatment-phase synthesis; when such studies reported signatures explicitly linked to treatment response or outcome prediction, they were retained for descriptive analysis.

#### 2.2.4. Outcomes

The primary outcome was TB treatment response, defined by the original studies using microbiological, clinical, or composite endpoints (e.g., culture conversion, treatment success, treatment failure, relapse).

#### 2.2.5. Study Design

We included randomized and non-randomized study designs evaluating metabolomic biomarkers in relation to TB treatment response. Eligible designs included prospective or retrospective cohort studies, nested case–control studies within defined cohorts, randomized controlled trial (RCT) sub-studies, and cross-sectional analyses reporting treatment-stage case–control analyses were included when derived from well-defined clinical cohorts or longitudinal studies of TB treatment. Reviews, editorials, letters, case reports or case series, and conference abstracts without full-text publication were excluded.

### 2.3. Information Sources and Search Strategy

A systematic literature search was conducted in PubMed, Scopus, and EMBASE from inception to 26 November 2025. The search strategy combined controlled vocabulary and free-text terms related to tuberculosis, metabolomics, biomarkers, and treatment response. The search was limited to English-language publications.

To maximize sensitivity, search terms included variations of “tuberculosis”, “*Mycobacterium tuberculosis*”, “metabolomics”, “metabonomics”, “lipidomics”, “biomarker”, “treatment”, “therapy”, “response”, “monitoring”, “outcome”, “failure”, and “cure”. The full search strategy is provided in Supplementary Table S1. Reference lists of included studies and relevant reviews were also screened to identify additional eligible publications.

### 2.4. Study Selection

All records retrieved from database searches were exported to reference management software Mendeley Desktop (Version 1.19.8) and deduplicated prior to screening. Two reviewers independently screened titles and abstracts to identify potentially eligible studies. Full-text articles were then retrieved and assessed independently by the same reviewers against the predefined eligibility criteria (PECOS framework). Disagreements at any stage were resolved through discussion and consensus; when consensus could not be reached, a third reviewer was consulted. Inter-reviewer agreement was assessed qualitatively based on consistency in study inclusion decisions.

**Figure 1:**
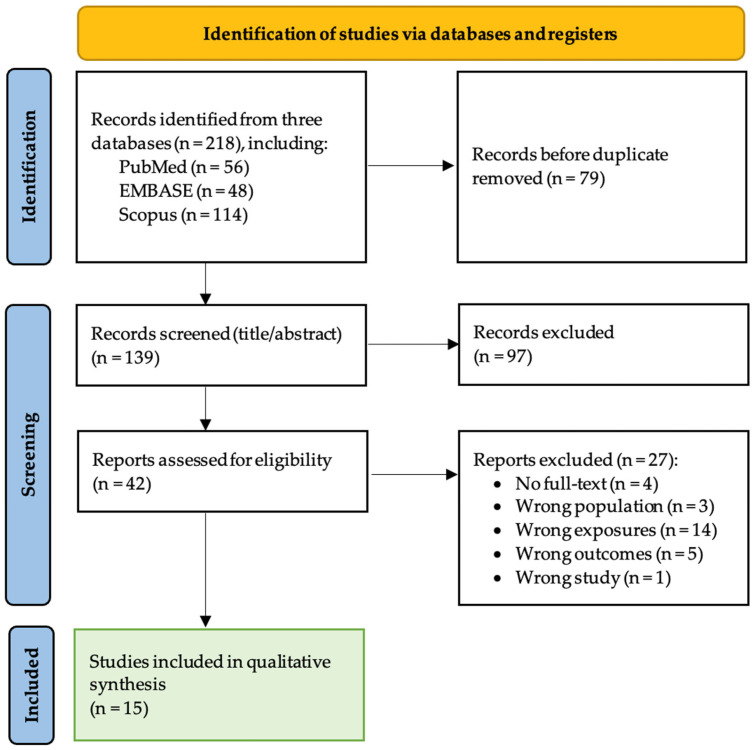
PRISMA flow diagram of study selection.

Reasons for full-text exclusion were documented. The study selection process is summarized using a PRISMA flow diagram (Figure 1).

### 2.5. Data Extraction

Data were extracted independently by two reviewers using a standardized extraction form, which was pilot-tested on a subset of studies to ensure consistency and clarity. Extracted variables included: author and year; country/setting; study design; participant characteristics (e.g., age group, HIV status, and comorbidities when reported); sample size; type of TB and diagnostic criteria; biological specimen (e.g., urine or plasma); metabolomics platform and analytical approach (targeted or untargeted); sampling time points during treatment; definition of treatment response and outcomes; statistical methods; and key reported metabolites or metabolite panels associated with treatment response. Where available, directionality of metabolite change (increase or decrease) was recorded for each comparison category.

Any discrepancies in extracted data were resolved by discussion and consensus between reviewers. When required, corresponding authors were contacted for clarification.

### 2.6. Risk of Bias Assessment

Risk of bias was assessed independently by two reviewers. Studies that developed, validated, or externally evaluated multivariable prediction models (including machine learning classifiers) were evaluated using the Prediction Model Risk Of Bias Assessment Tool (PROBAST). Observational studies reporting metabolite–outcome associations or longitudinal metabolite changes without a formal prediction model were evaluated using the Quality In Prognostic Studies (QUIPS) tool.

Studies were categorized as prognostic association studies or prediction model studies based on whether multivariable predictive models were developed and internally or externally validated. Disagreements were resolved by discussion and consensus.

For PROBAST, risk of bias was assessed across four domains (participants, predictors, outcome, and analysis), and applicability was assessed across three domains (participants, predictors, and outcome). For QUIPS, risk of bias was assessed across study participation, prognostic factor measurement, outcome measurement, confounding, and statistical analysis/reporting. Overall risk-of-bias ratings were derived using a conservative approach: studies were classified as high risk if any domain was rated as high risk; otherwise, studies were rated as unclear/moderate if any domain was unclear or moderate, and as low risk if all domains were rated low risk. This conservative approach is consistent with recommended practices for minimizing bias in systematic reviews of prognostic and prediction studies [27,28].

The use of these tools follows established guidance for prediction and prognostic studies [27,28].

### 2.7. Data Synthesis and Analysis

Due to substantial heterogeneity in study design, sampling time points, analytical platforms, metabolite identification, and statistical reporting, a quantitative meta-analysis was not performed. Instead, findings were synthesized narratively and summarized using a structured comparison framework. A pathway-level synthesis approach was used to integrate biologically related metabolites and improve interpretability across heterogeneous studies. A recurrence-based approach was used to identify consistent signals across studies, acknowledging the limitations of vote-counting methods, including lack of effect-size weighting and potential bias.

Comparisons were classified into four predefined groups: (Group 1) baseline vs. end of treatment; (Group 2) baseline vs. intensive phase; (Group 3) intensive phase vs. end of treatment; and (Group 4) treatment failure vs. cure.

Where reported, directionality of metabolite change (increase or decrease) was summarized within each comparison group. Metabolites were considered recurring when reported in at least two studies within the same comparison category, as a pragmatic threshold to identify consistent signals across heterogeneous datasets. However, this recurrence-based approach has limitations, including lack of effect-size weighting and potential bias toward frequently studied metabolites. Recurrence was defined within the same comparison category and biofluid. Results were further stratified by biological specimens (urine vs. plasma) and metabolomics approach (targeted vs. untargeted) when data permitted.

## 3. Results

### 3.1. Study Selection

The database search identified 218 records. After removal of duplicates, 139 records were screened at the title and abstract stage, of which 97 were excluded. A total of 42 full-text reports were retrieved and assessed for eligibility; 27 were excluded, most commonly because the studies did not evaluate metabolomic biomarkers in relation to TB treatment response. In total, 15 studies met the inclusion criteria and were included in the qualitative synthesis (Figure 1). These studies were conducted across nine countries and included a range of study designs, including prospective and retrospective cohort studies, nested case–control analyses, and prediction model studies.

### 3.2. Study Characteristics

Characteristics, biospecimens, and analytical platforms of the included studies are summarized in Table 1. Overall, the evidence base comprised heterogeneous study designs, including longitudinal cohort studies, nested case–control analyses, and prediction model studies. Studies were conducted across multiple geographic settings, including South Africa, Uganda, Ethiopia, Haiti, China, Thailand, India, Brazil, and Korea. Populations included adults and children, and several studies evaluated cohorts with key comorbidities such as HIV infection or diabetes mellitus.

### 3.3. Biospecimens and Metabolomic Platforms

Across the 15 included studies, metabolomic profiling was performed using both plasma and urine biospecimens. The majority of studies employed LC–MS-based workflows, while fewer used GC–MS or GC×GC–MS or UPLC platforms (Table 1). Both untargeted and targeted metabolomic approaches were represented, including targeted lipidomics and targeted quantification of immune-related metabolites such as markers within the tryptophan–kynurenine pathway.

### 3.4. Treatment Response Comparison Group

**Figure 2:**
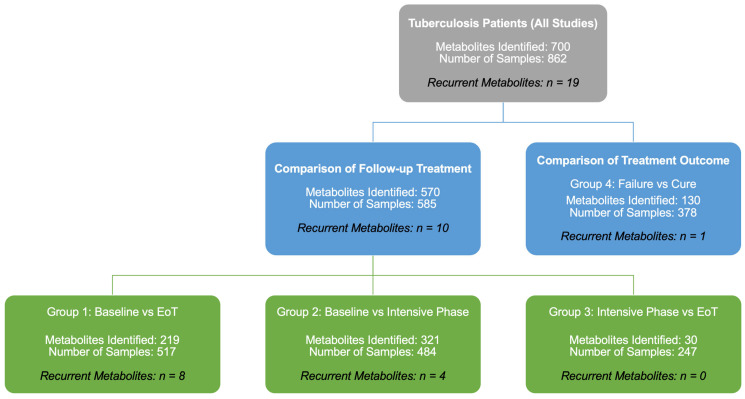
Study synthesis workflow and comparison-group framework used for narrative integration.

To facilitate synthesis across heterogeneous study designs and sampling schedules, included studies were categorized into four clinically relevant comparison groups (Figure 2): (1) baseline versus end of treatment (EoT), (2) baseline versus intensive phase, (3) intensive phase versus EoT, and (4) treatment failure versus cure outcome. This grouping enabled structured comparison of longitudinal treatment-associated metabolic changes as well as baseline metabolic signatures associated with unfavorable treatment outcomes.

The included studies were organized into four comparison groups based on treatment stage and outcome definitions: (Group 1) baseline vs. end of treatment (EoT), (Group 2) baseline vs. intensive phase, (Group 3) intensive phase vs. EoT, and (Group 4) treatment failure/relapse vs. cure. Metabolite-level findings were synthesized using a structured narrative approach with recurrence-based vote counting across studies within each comparison group. Quantitative effect-size meta-analysis was not performed due to heterogeneity in study designs, biospecimens, analytical platforms, sampling schedules, and reporting formats.

### 3.5. Summary of Metabolites by Comparison Group

**Figure 3:**
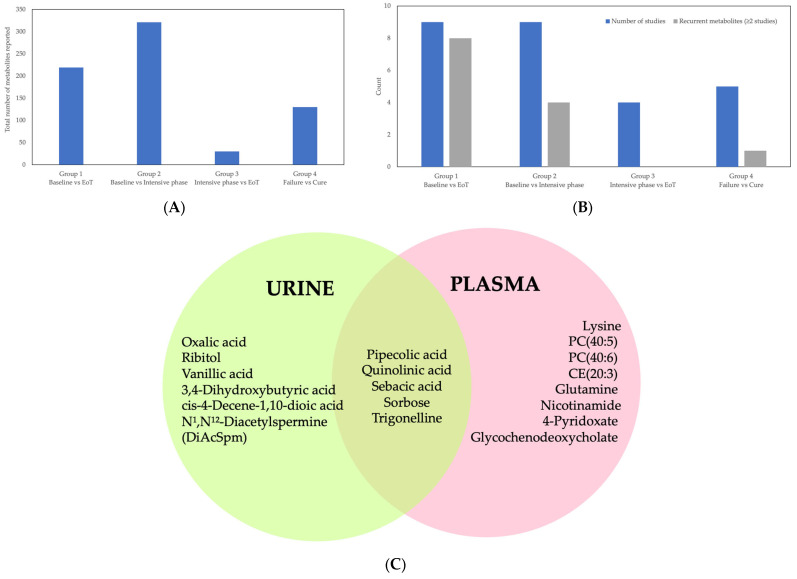
Summary of study distribution and recurrent metabolites across comparison groups and sample types. (**A**) Total number of metabolites reported across studies within each comparison group: Group 1 (baseline vs. end of treatment [EoT]), Group 2 (baseline vs. intensive phase), Group 3 (intensive phase vs. EoT), and Group 4 (treatment failure vs. cure); (**B**) Number of included studies contributing to each comparison group and the number of recurrent metabolites (defined as metabolites reported in ≥2 independent studies) within that group; (**C**) Venn diagram showing the overlap of recurrent metabolites (≥2 studies) identified in urine and plasma, highlighting biofluid-specific signatures and limited cross-matrix overlap.

A total of 700 metabolites were reported across all comparison groups (Table 2). Metabolite counts are not directly comparable across studies due to differences in analytical coverage, metabolite identification workflows, statistical thresholds, and reporting practices. Metabolite totals are not additive across groups because individual studies may contribute to multiple comparison categories and the same metabolite may be reported in more than one group. The distribution of studies and metabolites by comparison group is summarized in Figure 3A,B, and recurrent metabolites within each subgroup are summarized in Table 2.

Group 1 (baseline vs. EoT) included nine studies reporting 219 metabolites, comprising 157 plasma metabolites across six studies and 62 urine metabolites across three studies (Table 2). Eight metabolites were recurrent across ≥2 studies in this group, including 4-pyridoxate, glutamine, glycochenodeoxycholate, lysine, N^1^,N^12^-Diacetylspermine (DiAcSpm), nicotinamide, quinolinic acid, and trigonelline. Only N^1^,N^12^-Diacetylspermine (DiAcSpm) was identified across urine studies in this group.

Group 2 (baseline vs. intensive phase) included nine studies reporting 321 metabolites, comprising 138 plasma metabolites across five studies and 183 urine metabolites across four studies (Table 2). Four recurrent metabolites were identified in this group, including 4-pyridoxate, glycochenodeoxycholate, nicotinamide, and trigonelline.

Group 3 (intensive phase vs. EoT) included four studies reporting 18 plasma metabolites and 12 urine metabolites, with no recurrent metabolites identified across studies (Table 2).

Group 4 (treatment failure vs. cure) included five studies reporting 130 metabolites, comprising 62 plasma metabolites from one study and 68 urine metabolites from four studies (Table 2). One recurrent urine metabolite, cis-4-decene-1,10-dioic acid, was reported in ≥2 studies, while no recurrent plasma metabolites were identified.

Across comparison groups, overlap between plasma and urine metabolite findings was limited, indicating that metabolomic treatment-response signatures were strongly biofluid-dependent (Figure 3C).

### 3.6. Risk of Bias Assessment

Risk of bias assessments are summarized in Table 3. Prediction model studies (including machine learning classifiers) were assessed using PROBAST, while prognostic association studies were assessed using QUIPS. Overall, seven studies were classified as prediction model studies and eight studies as prognostic association studies.

Among the seven prediction model studies assessed using PROBAST, six were judged to have a high risk of bias, while one study was judged low risk of bias (Table 3A). The most frequent PROBAST concerns were within the analysis domain, driven by small sample sizes relative to model complexity, high overfitting risk, incomplete reporting of model development steps, and limited internal or external validation.

Among the eight prognostic association studies assessed using QUIPS, three were judged low risk of bias, three moderate risk, and two high risk (Table 3B). The most common QUIPS concerns were related to confounding control (particularly incomplete adjustment for HIV status, diabetes mellitus, and drug resistance), and analysis/reporting, including incomplete reporting of missing data handling and variability in outcome definitions and sampling schedules across cohorts.

Given these methodological limitations, particularly heterogeneity in analytical workflows and the high risk of bias in most prediction model studies, we therefore focused the synthesis on recurrent pathway-level signals that were consistently observed across independent cohorts and study designs.

### 3.7. Metabolic Pathway Synthesis

Across comparison groups, recurrent metabolites converged on four principal pathway domains: (i) amino acid metabolism, particularly the tryptophan–kynurenine axis; (ii) vitamin and cofactor metabolism; (iii) lipid remodeling and bile acid metabolism; and (iv) polyamine and β-oxidation–related pathways.

In longitudinal treatment comparisons (Groups 1–3), the most consistent signals involved treatment-associated modulation of the tryptophan–kynurenine pathway and vitamin/cofactor metabolites, reflecting shifts in systemic immune activation and redox balance during therapy. Plasma-based analyses repeatedly highlighted the kynurenine/tryptophan ratio, quinolinic acid, pyridoxate, and nicotinamide as representative markers of metabolic recovery.

In contrast, baseline comparisons linked to unfavorable outcomes (Group 4) more frequently implicated lipidomic perturbations, including altered ceramides, sphingomyelins, and cholesteryl esters, suggesting that host lipid remodeling and inflammatory lipid mediators may contribute to risk stratification.

Urine-based studies highlighted polyamine metabolism (notably N^1^,N^12^-diacetylspermine) and dicarboxylic acids associated with β-oxidation, supporting the concept that urinary metabolites reflect downstream excretory and microbiome-associated metabolic processes distinct from plasma immune–metabolic signatures.

**Figure 4:**
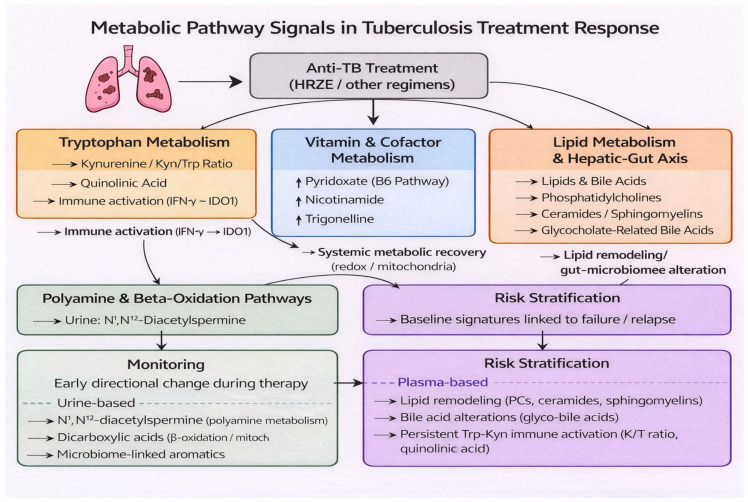
Metabolic pathway signals in tuberculosis treatment response. Schematic overview of recurrent metabolic pathways and representative metabolites associated with tuberculosis (TB) treatment response, synthesized from the 15 included studies and pathway-level findings (Table 2 and Table 4). Anti-TB therapy (HRZE or other regimens) is associated with coordinated immunometabolic changes across multiple biological domains. The most consistently reported pathway was tryptophan catabolism through the kynurenine axis, reflecting immune activation (IFN-γ–IDO1). Vitamin and cofactor metabolites (e.g., pyridoxate, nicotinamide, trigonelline) suggest systemic metabolic recovery during therapy. Lipid remodeling and bile acid perturbations reflect host lipid regulation and potential hepatic–gut axis influences. Urine-based studies additionally highlighted polyamine metabolism (e.g., N^1^,N^12^-diacetylspermine) and β-oxidation and excreted end-products, including dicarboxylic acids and microbiome-linked aromatics. The lower panels illustrate two potential translational applications: (i) longitudinal monitoring based on early directional changes during treatment and (ii) baseline risk stratification using metabolite signatures linked to failure/relapse. Solid arrows indicate direct or directional relationships between biological processes, while dashed lines represent indirect or inferred associations across pathways. Arrows indicate conceptual relationships rather than quantitative effect sizes.

Pathway-level findings extracted from individual studies are summarized in Table 4, and an integrated schematic distinguishing monitoring-related versus risk-stratification–related metabolic signals is presented in Figure 4.

## 4. Discussion

This review synthesizes evidence from 15 human studies evaluating metabolomic biomarkers for monitoring tuberculosis (TB) treatment response, encompassing longitudinal plasma- and urine-based analyses, lipidomic investigations, adult and pediatric populations, and outcome-focused prognostic and prediction model studies [16,17,18,19,20,21,22,23,24,25,26,27,28,29,30]. Across studies, anti-TB therapy was consistently associated with metabolic perturbations; however, the specific metabolites reported varied substantially depending on the biospecimen, analytical platform, sampling schedule, and reporting practices. By organizing findings according to treatment-stage and outcome comparisons (Figure 2) and prioritizing cross-study recurrence rather than single-study signals, this review identifies a limited set of metabolites and pathways that show convergent evidence of change during therapy, while also highlighting key methodological limitations that currently constrain direct clinical translation [11,12,33,34].

Across comparison groups, the most consistent treatment-response signal involved amino acid metabolism, particularly the tryptophan–kynurenine pathway. Multiple plasma-based studies reported dynamic changes in the kynurenine/tryptophan (K/T) ratio and downstream metabolites, such as quinolinic acid, across treatment time points [16,17,21,22,23,24,27,28]. In general, elevated K/T ratios and quinolinic acid levels were observed at baseline in active TB and declined during successful therapy, with several studies reporting treatment-associated reductions consistent with resolution of systemic immune activation. Conversely, persistent elevation of these markers was associated with unfavorable outcomes in treatment-outcome comparisons (Group 4; Table 2) and in prediction-oriented analyses [17,22,23,27]. These findings are biologically plausible, as interferon-γ-driven activation of indoleamine 2,3-dioxygenase promotes increased tryptophan catabolism during chronic infection and sustained immune activation [35,36,37,38]. The consistent observation of this pathway across studies conducted in different TB patient populations, including adult and pediatric cohorts, and populations with major comorbidities such as HIV infection and diabetes mellitus, suggests that the tryptophan–kynurenine axis reflects a recurrent host immunometabolism response during TB treatment rather than a cohort-specific phenomenon [18,19,22,25]. Nevertheless, because this pathway is not TB-specific and may be influenced by other inflammatory conditions, its most appropriate clinical role is likely within a multimetabolite panel rather than as a stand-alone biomarker [10,37,39,40].

It is important to distinguish between recurrent signals supported across multiple independent cohorts and exploratory findings derived from smaller or higher-risk studies. The tryptophan–kynurenine pathway represents the most robust and consistently replicated signal, whereas several lipidomic and urinary metabolites remain exploratory and require further validation.

In addition to amino acid metabolism, vitamin- and cofactor-related metabolites emerged as recurrent features of treatment response. Pyridoxate (a vitamin B6 catabolite), nicotinamide, and trigonelline were repeatedly reported in longitudinal comparisons, particularly between baseline and intensive-phase or end-of-treatment time points (Table 2) [16,21,22,23,24]. Across studies, these metabolites generally showed directional changes consistent with metabolic recovery during therapy, although the magnitude and timing of change varied across cohorts and analytical platforms. These metabolites plausibly reflect recovery of host nutritional status, redox balance, and mitochondrial function, which are disrupted during active TB and may progressively normalize with effective therapy [5,11,12,33]. Although the direction and timing of change were not fully consistent across studies, repeated detection of these metabolites across independent cohorts supports their potential relevance as markers of systemic metabolic recovery during treatment.

Lipid and bile acid metabolism constituted another prominent theme, particularly in plasma-based studies and analyses focused on treatment outcomes. Lipidomic profiling identified treatment-associated remodeling of phosphatidylcholines and sphingolipids, while baseline lipid signatures, including altered ceramide and sphingomyelin profiles, were associated with subsequent treatment failure in some cohorts [17,23,24,27]. Recurrent alterations in bile acids such as glycochenodeoxycholate and glycocholate were also reported [21,23], potentially reflecting interactions among host metabolism, antimicrobial therapy, hepatic function, and gut–liver axis biology [41,42]. However, interpretation of lipid and bile acid markers is subject to important confounding factors. These metabolites are highly sensitive to dietary intake, liver function, and antibiotic exposure, all of which can substantially influence circulating and excreted metabolite levels independent of TB disease activity or treatment response. In addition, variability in hepatic function, whether related to underlying disease, drug-induced hepatotoxicity, or nutritional status, may further affect bile acid metabolism and lipid profiles. Therefore, lipid and bile acid markers are sensitive to diet, liver disease, and antibiotic exposure, and may therefore be most informative when interpreted in combination with other metabolic and clinical indicators [43,44,45,46,47].

Urine-based metabolomic studies highlighted additional pathways relevant to treatment monitoring, particularly polyamine metabolism and fatty acid β-oxidation. Urinary N^1^,N^12^-diacetylspermine was one of the few metabolites consistently associated with early treatment response and changes in bacterial burden across multiple studies (Table 2) [16,20,21]. In most reports, levels of this metabolite declined during therapy, consistent with reduced inflammatory and proliferative metabolic activity as treatment progresses. Other urinary markers, including dicarboxylic acids and aromatic compounds, were linked to unfavorable outcomes and were hypothesized to reflect impaired mitochondrial β-oxidation or host-microbial metabolic interactions [25,30]. The limited overlap between urine and plasma metabolites observed in Figure 3C underscores that treatment-response signatures are strongly biofluid-dependent. Plasma appears to better capture systemic immune-metabolic changes and lipid remodeling, whereas urine reflects excreted metabolic end-products and polyamine-related immune-metabolic signals [12,48,49,50]. These findings emphasize the importance of defining the intended clinical use case, systemic monitoring versus non-invasive screening, when developing metabolomic biomarkers for TB treatment response.

From a translational perspective, the findings summarized in Figure 3 and Figure 4 and Table 2 and Table 3 suggest two potential applications of metabolomic biomarkers in TB. First, longitudinal markers that demonstrate early and reproducible directional change during therapy may support adjunctive monitoring of treatment response, particularly in settings where microbiological assessments are delayed, limited, or unavailable [16,20,21,23,24]. Second, baseline metabolic signatures associated with later treatment failure raise the possibility of pre-treatment risk stratification to guide intensified monitoring or tailored interventions [17,23,24,27]. However, reported performance metrics varied widely across studies, external validation was uncommon, and several prediction models were developed in relatively small cohorts. In addition, calibration performance was rarely assessed, and formal evaluation of clinical utility (e.g., decision-curve analysis) was generally lacking. These limitations indicate that most proposed biomarker panels remain investigational and are not yet suitable for clinical implementation [10,11,12,50].

Based on recurrence and biological plausibility, the most promising candidates for translational validation include the kynurenine/tryptophan ratio, quinolinic acid, pyridoxate, nicotinamide, and N^1^,N^12^-diacetylspermine, which collectively capture immune activation, metabolic recovery, and host response dynamics.

For metabolomics-based biomarkers to become clinically actionable in TB treatment monitoring, the most realistic near-term approach is likely a targeted quantitative assay rather than untargeted discovery workflows. In practice, this would most plausibly take the form of a targeted LC–MS/MS panel measuring a small number of recurrent metabolites (e.g., 3–6 markers), selected to represent complementary biological domains such as immune activation (Trp–Kyn markers), metabolic recovery (pyridoxate, nicotinamide), and host lipid remodeling (selected lipid or bile acid features) [11,37,40,42,43,45,47]. At present, such panels should be considered exploratory and hypothesis-generating, pending rigorous external validation, calibration assessment, and demonstration of clinical utility in prospective studies. Such panels are technically feasible, potentially cost-effective, and compatible with standardized calibration procedures, quality control frameworks, and inter-laboratory reproducibility.

Plasma-based assays may be better suited for capturing systemic immune–metabolic shifts and lipid remodeling, whereas urine-based assays offer advantages in non-invasive sampling and feasibility for longitudinal monitoring in resource-limited settings. However, given the limited overlap between urine and plasma metabolites (Figure 3C), plasma and urine panels should be developed for distinct clinical applications rather than assumed to be interchangeable. Urine-based candidates such as N^1^,N^12^-diacetylspermine remain particularly attractive for early treatment monitoring, but require additional validation across diverse cohorts and standardized normalization strategies [16,20,21].

Across both matrices, translation will require (i) standardized biospecimen collection and processing protocols, (ii) clear reporting of metabolite identification confidence (e.g., MSI levels), (iii) harmonized clinical endpoints and sampling schedules aligned with treatment milestones, (iv) prospective multicenter external validation using pre-specified panels rather than post hoc feature selection, and (v) standardized inclusion and exclusion criteria that account for populations vulnerable to metabolic variability, including individuals with HIV infection, diabetes mellitus, or pediatric TB [5,11,12,31,48]. Without these steps, reported biomarkers are likely to remain context-specific and vulnerable to poor reproducibility across settings.

Several sources of heterogeneity complicate interpretation of the existing evidence. Differences in metabolite identification confidence (e.g., Metabolomics Standards Initiative (MSI) levels) further contribute to heterogeneity across studies. Included studies differ substantially in analytical platforms, metabolite identification confidence, preprocessing pipelines, statistical methods, sampling schedules, and definitions of treatment response [5,11,12,31]. Many studies excluded or incompletely accounted for key comorbidities such as HIV infection or diabetes mellitus, despite their known influence on metabolic pathways repeatedly implicated in this review [19,22]. Risk-of-bias assessment (Table 3) identified common concerns related to participant selection, confounding control, and analytical transparency, particularly in prediction model studies [51,52]. In addition, several studies used nested case–control designs within larger cohorts, which may introduce spectrum or selection bias and potentially inflate biomarker performance estimates compared with fully prospective cohort analyses. These limitations underscore the need for standardized metabolomic workflows, harmonized outcome definitions, and rigorous validation strategies.

Importantly, heterogeneity across studies arises from multiple sources, including biospecimen type (plasma vs. urine), analytical platform (LC–MS, GC–MS, NMR), study populations (age, HIV status, metabolic comorbidities), and endpoint definitions. These factors directly influence metabolite detectability, biological interpretation, and comparability across studies, and should be carefully considered in future validation efforts.

This review has several strengths, including comprehensive synthesis of longitudinal and outcome-focused metabolomic studies, structured subgroup analyses aligned to treatment stages and biospecimens (Figure 2 and Figure 3), and pathway-level integration of recurrent findings (Figure 4; Table 4). Limitations include the inability to perform quantitative effect-size meta-analysis due to heterogeneity, reliance on recurrence-based synthesis rather than pooled estimates, and the potential for publication bias. Accordingly, the metabolites and pathways highlighted here should be interpreted as candidate signals rather than definitive clinical biomarkers [5,11,12,31].

In summary, metabolomic profiling captures reproducible immunometabolic perturbations during TB therapy, with convergent evidence implicating the tryptophan–kynurenine axis, vitamin/cofactor metabolism, lipid remodeling, and urine polyamine-related pathways in treatment response. While these findings support the biological plausibility of metabolomics-based monitoring, substantial methodological heterogeneity and limited validation currently preclude routine clinical implementation. Prospective, multicenter studies using standardized protocols, harmonized outcome definitions, and rigorous validation frameworks will be essential to translate these promising metabolic signatures into clinically actionable tools for monitoring TB treatment response [5,11,12,31].

## 5. Conclusions

This review synthesizes current evidence on metabolomic biomarkers associated with TB treatment response. Across 15 human studies, reproducible but heterogeneous metabolic perturbations were observed during therapy, with convergent evidence implicating the tryptophan–kynurenine pathway, vitamin/cofactor metabolism, lipid remodeling, and urine polyamine-related pathways. While metabolomic profiling captures biologically meaningful immunometabolic changes during TB therapy, current evidence remains heterogeneous and insufficiently validated for routine clinical implementation.

These limitations reflect substantial heterogeneity in biospecimens, analytical platforms, sampling schedules, and outcome definitions, together with limited external validation. Future studies should prioritize standardized metabolomic workflows, harmonized definitions of treatment response, and prospective multicenter validation across key populations, including individuals with HIV infection and metabolic comorbidities. Such efforts will be essential to translate promising metabolomic signatures into clinically actionable tools for monitoring TB treatment response.

## Figures and Tables

**Table 1:** Characteristics, biospecimens and metabolomic platforms used in included studies.

Author, Publication Year [Ref]	Setting and Population	Study Design	Biospecimen	Platform and Approach	Sample Size		Sampling Time Points	Metabolite Focus	Outcome Comparison
					TB	Non-TB			
Luies L et al., 2017 [30]	Adults with pulmonary TB; South Africa	Prospective longitudinal cohort	Urine	GC–MS/GC×GC–MS, Untargeted	41	0	Baseline	Global metabolites	Group 4
Anh NK et al., 2024 [24]	Adults with pulmonary TB; diabetes (17.1%); Korea	Prospective observational longitudinal cohort	Plasma	LC–MS, Untargeted & targeted	41	0	Baseline; intensive (between week 6 & 11); EoT (between week 18 & 26)	Polar and bile acids/lipids	Group 1 and Group 2
Anh NK et al., 2023 [23]	Adults with pulmonary TB; diabetes (17.1%); Korea	Longitudinal cohort study	Plasma	LC–MS, Targeted lipidomics	41	0	Baseline; intensive (between week 6 & 11); EoT (between week 18 & 26)	Lipids	Group 1 and Group 2
Fitzgerald BL et al., 2019 [26]	Adults with pulmonary TB; HIV (-)^1^; Multi-center (Uganda & South Africa)	Uganda cohort (KCHS): Cohort subset/case-contact study South Africa cohort (Catalysis Study): Longitudinal outcome cohort	Urine	LC–MS, Untargeted	45	39	Baseline; week 1/2/4/8, EoT	Global metabolites	Group 2 and Group 4
Dutta NK et al., 2020 [28]	Children with pulmonary and extrapulmonary TB; India	Longitudinal nested case–control study (within the CTRIUMPH cohort)	Plasma	LC–MS/MS, Untargeted	16	16	Baseline; month 1; month 6 (EoT)	Polar metabolites	Group 1 and Group 2 and Group 3
Combrink M et al., 2019 [20]	Adults with pulmonary TB; South Africa	Prospective longitudinal pharmacometabolomics study	Urine	GC×GC–TOFMS, Untargeted	23	0	Baseline; week 1/2/4	Global metabolites	Group 2
Collins JM et al., 2025 [17]	Adults with pulmonary TB; Ethiopia	Case–control + longitudinal follow-up	Plasma	LC–MS (panel) & ML, Targeted & ML	82	104	Baseline; Month 2/6/12 after treatment	153-metabolite panel	Group 1 and Group 2 and Group 3
Xia Q et al., 2020 [21]	Adults with pulmonary TB; HIV (Africa 11.8% & Haiti 0%); Multi-center (Africa & Haiti)	Prospective longitudinal cohort analysis (2 cohorts)	Urine	LC–MS & ELISA, Targeted	69	0	Baseline; weeks 2/4/8/17/26; week 52 post-treatment)	DiAcSpm	Group 1 and Group 4
Shivakoti R et al., 2022 [27]	Adults with pulmonary TB; diabetes (32%), HIV (2%); India	Case–control study, nested within a prospective cohort	Plasma	LC–MS, Untargeted	192	0	Baseline	Global metabolites	Group 4
Mahapatra S et al., 2014 [16]	Adults with pulmonary TB; HIV (-) ^1^; Multi-center (Uganda & South Africa)	Prospective observational cohort of TB patients with longitudinal treatment sampling	Urine	LC–MS, Untargeted	87	0	Baseline; month 1/2/6	Global metabolites	Group 1 and Group 2
Gatechompol S et al., 2024 [19]	Adults with pulmonary TB; HIV (+); Thailand	Nested case–control (within prospective HIV cohort on ART)	Plasma	LC–MS/MS, Targeted	13	13	Pre-TB (6 months before TB diagnosis); diagnosis (baseline); EoT (6 months after TB treatment)	Tryptophan–Kynurenine pathway	Group 1 and Group 4
Yang et al., 2024 [22]	Adults with pulmonary TB; type 2 diabetes mellitus (50%), HIV (-)^1^; China	Prospective cohort (targeted metabolite quantification)	Plasma	UPLC–MRM, Targeted	32	32	Baseline; month 6 of post-treatment	Quinolinic acid panel	Group 1
Luies L et al., 2017 [29]	Adults with pulmonary TB; South Africa	Prospective observational cohort study	Urine	GC×GC–TOFMS, Untargeted	31	0	Baseline	Global metabolites	Group 4
Tornheim JA et al., 2022 [18]	Children with pulmonary and extrapulmonary TB; HIV (-)^1^; India	Targeted diagnostic accuracy analysis (secondary analysis from cohort biorepository)	Plasma	LC–MS/MS, Targeted	16	32	Baseline; month 1; EoT	Tryptophan–Kynurenine pathway	Group 1 and Group 2
Arriaga MB et al., 2022 [25]	Adults with pulmonary TB; dysglycemia (31.1%), HIV (22.3%); Multi-center (Brazil & South Africa)	Prospective longitudinal cohort	Urine	UPLC–MS/MS, Targeted	133	60	Baseline; month 2; month 6 (EoT)	Eicosanoids	Group 1 and Group 2 and Group 3

^1^ HIV (-): Confirmed HIV-negative status; other comorbidities were not reported (NR). Specific Conditions: Listed with prevalence (%) where applicable (e.g., Diabetes (17.1%), HIV (2%)). Abbreviations: DiAcSpm, N^1^,N^12^-Diacetylspermine; ELISA, enzyme-linked immunosorbent assay; EoT, end of treatment; GC, gas chromatography; Group 1, Baseline vs. EoT; Group 2, Baseline vs. intensive phase; Group 3, Intensive phase vs. EoT; Group 4, Treatment failure vs. cure; LC, liquid chromatography; ML, machine learning; MRM, Multiple Reaction Monitoring; MS, mass spectrometry; MS/MS, tandem mass spectrometry; TOF, time of flight; TB, tuberculosis; UPLC, ultra performance liquid chromatography.

**Table 2:** Summary of recurrent metabolites by treatment-response comparison group and biospecimen.

Comp. Group	Definition	Biospecimen	No. Studies	Total Metabolites	No. of Recurrent Metabolites	Recurrent Metabolites (≥2 Studies) *	Predominant Direction During Successful Therapy	Supporting Studies [Ref]
Group 1	Baseline vs. EoT	Plasma	6	157	7	4-Pyridoxate; Glutamine; Glycochenodeoxycholate; Lysine; Nicotinamide; Quinolinic acid; Trigonelline (N’-methylnicotinate)	Mostly decreased from baseline to EoT (normalization)	[17,18,22,24,28]
		Urine	3	62	1	N^1^,N^12^-Diacetylspermine (DiAcSpm)	Mostly decreased from baseline to EoT (normalization)	[16,21,25]
Group 2	Baseline vs. intensive phase	Plasma	5	138	4	4-Pyridoxate; Glycochenodeoxycholate; Nicotinamide; Trigonelline (N′-methylnicotinate)	Mostly decreased early during intensive phase	[17,18,23,24,28]
		Urine	4	183	0	NR	—	[16,20,25,26]
Group 3	Intensive phase vs. EoT	Plasma	3	18	0	NR	—	[17,18,28]
		Urine	1	12	0	NR	—	[25]
Group 4	Treatment failure vs. cure	Plasma	1	62	0	NR	—	[27]
		Urine	4	68	1	cis-4-Decene-1,10-dioic acid	Higher in failure/non-response (unfavorable outcome)	[21,26,29,30]

* Recurrent metabolites were defined as those reported in ≥2 included studies within the same comparison group and biospecimen. Direction of change reflects the predominant trend reported across studies. Abbreviations: EoT, end of treatment; NR, no recurrent. Total metabolite counts represent unique reported metabolites extracted from each subgroup and are not directly comparable across studies due to differences in analytical coverage and reporting.

**Table 3:** Risk of bias assessment of included studies. Prediction model studies (including machine learning classifiers) were evaluated using PROBAST. Prognostic factor/association studies were evaluated using QUIPS. (**A**) Prediction model studies (PROBAST). (**B**) Prognostic factor/association studies (QUIPS).

(**A**)								
**Study (Year) [Ref]**	**Study Classification**	**Participants**	**Predictors**	**Outcome**	**Analysis**	**Overall RoB**	**Overall Applicability**	**Key Concern(s)**
Collins et al., 2025 [17]	Prediction model (external evaluation)	Low	Low	Low	Low	Low	Low	Externally evaluated; clear modeling
Nguyen Ky Anh et al., 2023 [23]	Prediction model/ML classifier	Unclear	Low	Low	High	High	Unclear	Small N vs. predictors; limited validation
Mahapatra et al., 2014 [16]	Prediction model/ML classifier	High	High	Unclear	High	High	Unclear	Feature-level reporting; unclear model handling
Luies et al., 2017 [30]	Prediction model/ML classifier	High	Low	Low	High	High	Unclear	Small N; overfitting risk
Dutta et al., 2020 [28]	Prediction model/ML classifier	Unclear	Unclear	Low	High	High	Unclear	Complex integrated analysis; limited validation
Shivakoti et al., 2022 [27]	Prediction model/ML classifier	Unclear	Unclear	Unclear	High	High	Unclear	Confounding; limited model reporting
Tornheim et al., 2022 [18]	Prediction model/ML classifier	Unclear	Unclear	Unclear	High	High	Unclear	Small cohort; limited performance reporting
(**B**)								
**Study (Year) [Ref]**	**Study Classification**	**Participation**	**Factor Measurement**		**Outcome Measurement**	**Confounding**	**Analysis/Reporting**	**Overall RoB**
Luies et al., 2017 [29]	Association/longitudinal metabolite study	Moderate	Moderate		Low	Moderate	Moderate	Moderate
Nguyen Ky Anh et al., 2024 [24]	Association/longitudinal metabolite study	Moderate	Low		Low	Moderate	Moderate	Moderate
Fitzgerald et al., 2019 [26]	Association/longitudinal metabolite study	Moderate	Low		Low	High	High	High
Combrink et al., 2019 [20]	Association/longitudinal metabolite study	High	Low		Moderate	Moderate	Moderate	High
Xia et al., 2020 [21]	Association/longitudinal metabolite study	Low	Low		Low	Low	Low	Low
Gatechompol et al., 2024 [19]	Association/longitudinal metabolite study	Moderate	Low		Moderate	Moderate	Moderate	Moderate
Yang et al., 2024 [22]	Association/longitudinal metabolite study	Low	Low		Low	High	High	High
Arriaga et al., 2022 [25]	Association/longitudinal metabolite study	Low	Low		Low	Low	Low	Low

Abbreviations: RoB, risk of bias; ML, machine learning. PROBAST was applied only to studies that developed, validated, or externally evaluated multivariable prediction models (including machine learning classifiers). QUIPS was applied to studies reporting metabolite–outcome associations without a formal prediction model.

**Table 4:** Key metabolites reported across studies, organized by biospecimen and comparison group. This table summarizes representative metabolites reported in at least one study within each subgroup.

Comparison Group	Plasma (Representative Metabolites)	Urine (Representative Metabolites)	Key Pathway Themes	Key Supporting Studies [Ref]	Clinical Relevance
Group 1 (baseline vs. EoT)	K/T ratio; quinolinic acid; nicotinamide; glutamine; glycochenodeoxycholate	N^1^,N^12^-diacetylspermine	Trp–Kyn; vitamin/cofactor; bile acids	[16,17,18,21,22,24,25,28]	Treatment monitoring
Group 2 (baseline vs. intensive)	K/T ratio; 4-Pyridoxate; nicotinamide; trigonelline; bile acids	NR; multiple unique features	Trp–Kyn; Early immunometabolic shift; polyamines; β-oxidation	[16,17,18,20,23,24,25,26,28]	Treatment monitoring
Group 3 (intensive vs. EoT)	K/T ratio	NR	Trp–Kyn; Late-phase metabolic normalization (heterogeneous)	[17,18,25,28]	Treatment monitoring
Group 4 (failure vs. cure)	NR	cis-4-Decene-1,10-dioic acid; aromatic metaboloties	Lipid remodeling; β-oxidation; microbiome-related aromatics	[21,26,27,29,30]	Risk stratification

Abbreviations: K/T: kynurenine/tryptophan; NR: no recurrent; Trp–Kyn: tryptophan–kynurenine.

## Data Availability

No new datasets were generated or analyzed during this study. All data analyzed in this review are derived from previously published studies cited in the manuscript.

## Supplementary Materials

The following supporting information can be downloaded at: https://www.mdpi.com/article/10.3390/diagnostics16091278/s1, Table S1. Full search strategy used for database searches.

## Abbreviations

The following abbreviations are used in this manuscript:

TB	Tuberculosis
LC	Liquid chromatography
LC-MS	Liquid chromatography–mass spectrometry
MS	Mass spectrometry
MS/MS	Tandem mass spectrometry
GC	Gas chromatography
GC-MS	Gas chromatography–mass spectrometry
GC×GC–MS	Two-dimensional gas chromatography time-of-flight
CE	Capillary electrophoresis
CE–MS	Capillary electrophoresis–mass spectrometry
NMR	Nuclear magnetic resonance
PRISMA	Preferred Reporting Items for Systematic Reviews and Meta-Analyses
PROSPERO	International Prospective Register of Systematic Reviews
RCT	Randomized controlled trial
GenAI	Generative artificial intelligence
PROBAST	Prediction Model Risk Of Bias Assessment Tool
QUIPS	Quality In Prognostic Studies
ELISA	Enzyme-linked immunosorbent assay
EoT	End of treatment
ML	Machine learning
MRM	Multiple Reaction Monitoring
TOF	Time of flight
GC×GC–TOFMS	Two-dimensional gas chromatography time-of-flight mass spectrometry
UPLC	Ultra performance liquid chromatography
K/T ratio	Kynurenine/tryptophan ratio
Trp–Kyn	tryptophan–kynurenine
