# Supplementary material for: Metabolomic Biomarkers for Monitoring Tuberculosis Treatment Response: A Comprehensive Literature Review
Source: Diagnostics (Basel). 2026 Apr 23;16(9):1278. doi: 10.3390/diagnostics16091278 (PMC13162725; doi:10.3390/diagnostics16091278)
Supplement: Supplementary file 1 [file diagnostics-16-01278-s001.zip › diagnostics-4241646-supplementary-Table S1.pdf]

Scopus

Date of Search: 26/11/2025

Number of results: 114

Line	Search string
1	LANGUAGE (English) TITLE-ABS-KEY ("tuberculos*" OR "Tb" OR "Mycobacterium tuberculosis")
2	LANGUAGE (English) TITLE-ABS-KEY ("Biomarker*" OR "Diagnostic marker*" OR "Host biomarker*" OR "Prognostic marker*" OR "human biospecimen*" OR "human biomarker*" OR "biospecimen*" OR "Bio sign*" OR "human blood" OR "human plasma" OR "human serum" OR "human urine" OR "human sputum" OR "human BAL" OR "human bronchoalveolar lavage")
3	**1 AND 2**
4	LANGUAGE (English) TITLE-ABS-KEY ("Metabolom*" OR "metabolomic profilling" OR "metabolomic sign*" OR "metabolomic profile" OR "Mass Spectrometry" OR "Nuclear Magnetic Resonance" OR "NMR" OR "GC-MS" OR "LC-MS" OR "CE-MS" OR "metabolite*" OR "metabolic fingerprint*" OR "Mass spectrometry-based metabolom*" OR "MS-Based Metabolom*" OR "Nuclear Magnetic Resonance-based metabolom*" OR "NMR-Based Metabolom*" OR "NMR spectroscopy" OR "nmr metabolomic*" OR "Liquid Chromatography-Mass Spectrometry" OR "Gas Chromatography-Mass Spectrometry" OR "Capillary Electrophoresis-Mass Spectrometry" OR "Liquid Chromatography-Mass Spectrometry-based metabolom*" OR "Gas Chromatography-Mass Spectrometry-based metabolom*" OR "Capillary Electrophoresis-Mass Spectrometry-based metabolom*" OR "GC-MS-based metabolom*" OR "CE-MS-based metabolom*" OR "LC-MS-based metabolom*")
5	**3 AND 4**
6	LANGUAGE (English) TITLE-ABS-KEY ("Treatment Outcome" OR "Treatment Respone*" OR "Treatment Monitor*" OR "Therapy Response*" OR "Treatment Fail*" OR "Time of Treatment" OR "clinical response*" OR "response to treatment" OR "response to therapy" OR "Therapeutic monitor*")
7	**5 AND 6**

Pubmed

Date of Search: 26/11/2025

Number of results: 56

Line	Search string
**1**	"Tuberculosis"[MeSH Terms]
**2**	"Tuberculosis, Pulmonary"[MeSH Terms]
**3**	"Mycobacterium tuberculosis"[MeSH Terms]
**4**	#1 OR #2 OR #3
**5**	"tuberculosis"[Text Word] OR "mycobacterium tuberculosis"[Text Word] OR "tb"[Text Word]
**6**	#4 OR #5
**7**	"Metabolomics"[MeSH Terms]
**8**	"Mass Spectrometry"[MeSH Terms]
**9**	"Magnetic Resonance Spectroscopy"[MeSH Terms] OR "Nuclear Magnetic Resonance, Biomolecular"[MeSH Terms]
**10**	#7 OR #8 OR #9
**11**	"metabolomic*"[Text Word] OR "metabolite*"[Text Word] OR "metabolome*"[Text Word] OR "metabolic sign*"[Text Word] OR "metabolic fingerprint*"[Text Word] OR "metabolic profil*"[Text Word]
**12**	"Mass Spectrometry"[Text Word] OR "mass spectrometry-based"[Text Word] OR Mass spectrometry-based metabolom*[Text Word] OR MS-based metabolom*[Text Word] OR "GC-MS"[Text Word] OR "LC-MS"[Text Word] OR "CE-MS"[Text Word] OR LC-MS-based metabolom*[Text Word] OR GC-MS-based metabolom*[Text Word] OR CE-MS-based metabolom*[Text Word] OR "Liquid Chromatography-Mass Spectrometry"[Text Word] OR "Gas Chromatography-Mass Spectrometry"[Text Word] OR "Capillary Electrophoresis-Mass Spectrometry"[Text Word]
**13**	"nuclear magnetic resonance"[Text Word] OR "NMR"[Text Word] OR "NMR spectroscopy"[Text Word] OR Nuclear Magnetic Resonance-based metabolom*[Text Word] OR NMR-based metabolom*[Text Word]
**14**	#11 OR #12 OR #13
**15**	**#10 OR #14**
**16**	**#6 AND #15**

**17**	"Treatment Outcome"[MeSH Terms]
**18**	"treatment outcome*"[Text Word] OR "treatment response*"[Text Word] OR "treatment fail*"[Text Word] OR "treatment monitor*"[Text Word] OR "clinical response*"[Text Word] OR "response to therapy"[Text Word] OR "therapy response*"[Text Word] OR "therapeutic response*"[Text Word] OR "therapeutic monitor*"[Text Word] OR "time of treatment"[Text Word]
**19**	**#17 OR #18**
**20**	**#16 AND #19**
**21**	"Biomarkers"[MeSH Terms]
**22**	"biomarker*"[Text Word] OR "biosignature*"[Text Word] OR "diagnostic marker*"[Text Word] OR "host biomarker*"[Text Word] OR "prognostic marker*"[Text Word] OR "biological marker*"[Text Word] OR "biospecimen*"[Text Word] OR "bio sign*"[Text Word] OR "human blood"[Text Word] OR "human plasma"[Text Word] OR "human serum"[Text Word] OR "human urine"[Text Word] OR "human sputum"[Text Word] OR "human BAL"[Text Word] OR "human bronchoalveolar lavage"[Text Word]
**23**	**#21 OR #22**
**24**	**#20 AND #23**

EMBASE

Date of Search: 26/11/2025

Number of results: 48

Line	Search string
1	'tuberculosis'/exp/mj OR 'mycobacterium tuberculosis'/exp/mj
2	'tuberculos*':ab,ti OR 'mycobacterium tuberculosis':ab,ti OR 'tb':ab,ti
**3**	**1 OR 2**
4	'biological marker'/exp/mj OR 'diagnostic marker'/exp/mj OR 'prognostic marker'/exp/mj
5	'biomarker*':ab,ti OR 'biological marker*':ab,ti OR 'biosignature*':ab,ti OR 'diagnostic marker*':ab,ti OR 'host biomarker*':ab,ti OR 'prognostic marker*':ab,ti OR 'biospecimen*':ab,ti OR 'bio sign*':ab,ti OR 'human blood':ab,ti OR 'human plasma':ab,ti OR 'human serum':ab,ti OR 'human urine':ab,ti OR 'human sputum':ab,ti OR 'human BAL':ab,ti OR 'human bronchoalveolar lavage':ab,ti
6	4 OR 5
**7**	**3 AND 6**
8	'metabolomics'/exp/mj OR 'metabolic fingerprinting'/exp/mj
9	'metabolomic*':ab,ti OR 'metabolite*':ab,ti OR 'metabolome':ab,ti OR 'metabolic sign*':ab,ti OR 'metabolic fingerprint*':ab,ti OR 'metabolomic profil*':ab,ti OR 'metabolomic sign*':ab,ti
10	'mass spectrometry'/exp/mj
11	'mass spectrometry':ab,ti OR 'mass spectrometry-based metabolom*':ab,ti OR 'ms-based metabolom*':ab,ti OR 'gc-ms':ab,ti OR 'lc-ms':ab,ti OR 'ce-ms':ab,ti OR 'liquid chromatography-mass spectrometry':ab,ti OR 'gas chromatography-mass spectrometry':ab,ti OR 'capillary electrophoresis-mass spectrometry':ab,ti OR 'liquid chromatography-mass spectrometry-based metabolom*':ab,ti OR 'gas chromatography-mass spectrometry-based metabolom*':ab,ti OR 'capillary electrophoresis-mass spectrometry-based metabolom*':ab,ti OR 'gc-ms-based metabolom*':ab,ti OR 'ce-ms-based metabolom*':ab,ti OR 'lc-ms-based metabolom*':ab,ti
12	'nuclear magnetic resonance spectroscopy'/exp/mj
13	'nuclear magnetic resonance':ab,ti OR 'nmr-based metabolomic*':ab,ti OR 'nuclear magnetic resonance-based metabolomic*':ab,ti OR 'nmr metabolomic*':ab,ti OR 'NMR spectroscopy':ab,ti OR 'NMR':ab,ti
14	8 OR 9 OR 10 OR 11 OR 12 OR 13
**15**	**7 AND 14**
16	'treatment outcome'/exp/mj
17	'treatment response':ab,ti OR 'treatment outcome':ab,ti OR 'treatment fail*':ab,ti OR 'treatment monitor*':ab,ti OR 'clinical response':ab,ti OR 'response to treatment':ab,ti OR 'response to therapy':ab,ti OR 'time of treatment':ab,ti OR 'therapy response*':ab,ti OR 'therapeutic monitor*':ab,ti
18	16 OR 17
**19**	**15 AND 18**
**20**	**19 AND english:la**
